# Systemic and Mucosal B and T Cell Responses Upon Mucosal Vaccination of Teleost Fish

**DOI:** 10.3389/fimmu.2020.622377

**Published:** 2021-02-16

**Authors:** Estefanía Muñoz-Atienza, Patricia Díaz-Rosales, Carolina Tafalla

**Affiliations:** Fish Immunology and Pathology Laboratory, Animal Health Research Centre (CISA-INIA), Madrid, Spain

**Keywords:** teleost fish, mucosal vaccination, systemic immune responses, B cells, T cells, immunoglobulins

## Abstract

The development of mucosal vaccines against pathogens is currently a highly explored area of research in both humans and animals. This is due to the fact that mucosal vaccines have the potential to best elicit protective responses at these mucosal surfaces, which represent the frontline of host defense, thus blocking the pathogen at its initial replication sites. However, in order to provide an efficient long-lasting protection, these mucosal vaccines have to be capable of eliciting an adequate systemic immune response in addition to local responses. In aquaculture, the need for mucosal vaccines has further practical implications, as these vaccines would avoid the individual manipulation of fish out of the water, being beneficial from both an economic and animal welfare point of view. However, how B and T cells are organized in teleost fish within these mucosal sites and how they respond to mucosally delivered antigens varies greatly when compared to mammals. For this reason, it is important to establish which mucosally delivered antigens have the capacity to induce strong and long-lasting B and T cell responses. Hence, in this review, we have summarized what is currently known regarding the adaptive immune mechanisms that are induced both locally and systemically in fish after mucosal immunization through different routes of administration including oral and nasal vaccination, anal intubation and immersion vaccination. Finally, based on the data presented, we discuss how mucosal vaccination strategies could be improved to reach significant protection levels in these species.

## Introduction

Vaccination is a cost-efficient method to prevent the development of infectious diseases in humans and animals. Vaccine design aims to achieve long-term protection against a specific antigen by inducing an antigen-specific memory response in cells of the adaptive immune system, B and T cells, that will respond faster and with greater strength to a second encounter with this antigen. For this, antigen-presenting cells (APCs) are involved in digesting and degrading the antigen to provide antigen-specific activation of T cells, both CD4^+^ helper T cells and CD8^+^ cytotoxic T cells, the latter with the capacity to destroy virus-infected or tumor cells. B cells act as APCs and can also present the antigen to T cells, receiving in return activation signals from T helper cells in thymus-dependent (TD) responses or being activated by the antigen itself in the case of thymus-independent (TI) antigens. Upon activation, B cells start a differentiation process that leads them to a plasmablast state and eventually to become plasma cells, cells with a greater capacity to secrete immunoglobulins (Igs). Secreted Igs are referred to as antibodies. Throughout this differentiation process, memory B cells are also generated, as do memory T cells, also generated during the activation of T cells. In order to rationally design effective vaccines, it is fundamental to understand how these elements implicated in generating adaptive immunity respond to different vaccine formulations (1, 2). In mammals, different subsets of memory T lymphocytes have been described, including central memory T cells that remain in lymph and blood, tissue-resident memory T cells that reside within the tissues, and effector memory T cells that circulate amongst blood and peripheral tissues (3). In this context, CD4 tissue-resident memory T cells seem to play a key role in maintaining long-term protective immunity to mucosal pathogens as demonstrated in mice mucosally immunized with intranasal live attenuated vaccines against influenza virus (4) and coronaviruses (5). Regarding memory B lymphocytes, they are mainly found in the spleen and lymph nodes in mice and human, but can be also maintained as tissue-resident memory B cells at the sites of infection (6). Interestingly, intranasal live-attenuated influenza vaccines not only induced hemagglutinin-reactive plasmablasts but also memory B cells in the mediastinal lymph nodes (7). Therefore, the capacity of antigens to induce the generation of pathogen-specific tissue-resident memory B and T cells at mucosal surfaces seems as an important point to take into account when designing novel mucosal vaccines.

Most of the commercially available vaccines for both humans and animals are delivered through injection. However, in the past years, the interest and demand for the development of mucosal vaccines has considerably grown, taking into account that most infectious agents colonize the host through mucosal surfaces and it is at these sites that the initial replication steps occur (8). In this respect, having established that mucosal immunization has a superior ability to induce mucosal immune responses than systemic immunization (9), the search for effective mucosal vaccines is not only prompted from a logistic point of view, but also to produce vaccines that can efficiently block the pathogens at these early replicating sites before they are further disseminated throughout the organism. Despite all these advantages, there are only a few commercially available mucosal vaccines for humans or animals. This highlights the many challenges that the development of effective mucosal vaccines encounter. On one side, the vaccine formulations have to be correctly absorbed at the proper mucosal surface and taken up by a mucosal cell that can efficiently present the antigen. On the other hand, a successful mucosal vaccine has to overcome the mucosal tolerance that tightly controls the mucosal immune system to avoid unwanted immune responses to commensals or food antigens (8).

Despite these difficulties, some mucosal vaccines have shown to overcome mucosal tolerance and be able to generate efficient systemic immune responses in addition to local responses, including the production of specific antibodies and cell-mediated immunity (10–12), being this the basis for their efficacy, generating high levels of protection in humans. This is the case of the attenuated oral vaccine for the prevention of typhoid fever, caused by *Salmonella enterica* serovar Typhi, which elicits cell-mediated immune responses, involving both CD4^+^ helper T lymphocytes and CD8^+^ cytotoxic T cells (13). It is important to mention that although CD8^+^ T lymphocytes are mainly associated with defense against viral infections, these cells also play an important role clearing intracellular bacterial infections (14). Along the same line, the killed bivalent whole-cell *Vibrio cholerae* O1 and O139 vaccine produces a high increase of antibody titers and a strong O antigen–specific B memory response after oral immunization (15). In the case of mucosal vaccines against viruses, the intranasal administration of inactivated and live-attenuated influenza vaccines induce protective antibodies capable of inhibiting hemagglutination in serum (16). In this case, however, only the live-attenuated and not the inactivated vaccine generated influenza-specific CD4^+^ and CD8^+^ T cells (16). Additionally, the protective immune response to two oral live-attenuated rotavirus vaccines composed of a monovalent attenuated human rotavirus or five bovine–human rotaviruses is mostly due to rotavirus-specific immunoglobulin A (IgA) in serum (17). Oral vaccination has been also revealed successful to control infectious diseases in wildlife, including an oral vaccine against rabies used in foxes (18) and one against classical swine fever (CSF) administered to wild boar (19). Therefore, it seems that when mucosal vaccines are capable of inducing strong systemic adaptive immune effects, protection can be achieved.

In aquaculture, most commercially available vaccines are administrated by intraperitoneal injection, often providing a strong and long-lasting protection, especially in the case of antibacterial vaccines. However, this method involves fish handling which provokes a great amount of stress to the fish, which at the end results in reduced responsiveness to the vaccine and even in some unwanted mortalities. Furthermore, vaccination cannot be administered by injection at very early life stages, and it is precisely in these stages when fish are more susceptible to infectious diseases (20–22). For this reason, huge efforts are focused to find new approaches of mass vaccine administration through mucosal surfaces (*e.g*., oral immunization and immersion) as these routes have benefits from both an economic/practical and an animal welfare point of view (20–22).

Teleost fish lack well-organized lymphoid structures in their mucosal associated lymphoid tissues (MALTs) such as Peyer’s patches in the intestine or tonsils and adenoids in the nasal cavity but instead present diffuse immune cells scattered throughout the tissue. Although some authors accurately point out that this does not constitute a real MALT, for clarity, we will designate these immune cells as MALT as referred to in most fish immunology papers (23–25). In order to succeed in the development of mucosal vaccination for aquacultured fish, it is imperative that we understand how the regulatory constituents of the local immune system in teleost fish guarantee that antigens presented in MALTs produce appropriate systemic immune responses. In addition, teleost fish B cell responses also differ in many aspects from mammalian B2 cell (conventional B cell) responses, as fish B cells retain many innate features such as a strong phagocytic capacity and microbicidal activity (26), also described in mammalian innate B1 lymphocytes (27).

Teleost fish only express three antibody isotypes (IgM, IgD, and IgT/Z). Thus, fish B cells have never been shown to undergo class switch recombination, a process through which mammals replace the constant region of IgM by that of IgA, IgE, or IgG, antibodies with higher affinity and different effector functions (28, 29). In fact, in mammalian mucosal surfaces, it is IgA that plays a predominant immune role (30). Thus, in teleost fish, the majority of naïve B cells from central immune organs co-express IgM and IgD on the cell membrane, resembling naïve B cell subsets present in mammals (31). When these cells are activated, as in mammals, IgD is lost from the cell membrane, giving the rise to activated IgM^+^ B cells that have a plasmablast/plasma cell profile (32). However, unlike mammals, these cells do not differentiate further to IgA-secreting plasma cells. Given the lack of IgA in fish, the fish-specific IgT has been proposed as a functional equivalent of IgA based on recent findings. IgT^+^ B cells, which represent an independent B cell lineage, are more abundant than IgM^+^ B cells in mucosal surfaces such as the gut (33), the skin (34), and the gills (35), secreting polymeric IgT into the mucus to protect these tissues from pathogens. Finally, when fish were exposed to specific parasitic infections, IgT responses were shown to be predominant in the mucosal compartments in contrast to IgM responses that seemed restricted to systemic compartments (33–35). Despite this, systemic IgT responses have also been reported to both mucosal and systemic stimulations (36, 37), therefore the precise role of IgT in fish adaptive immunity is still largely unknown.

Interestingly, in mammals, some cells have been shown to undergo an unconventional class switch through which only IgD is produced (38, 39). These cells, that can secrete IgD, have been reported mainly in the upper respiratory tract (38, 39). Despite their identification, the precise role of IgD in mammals is still unknown. Thus far, circulating IgD has been shown to interact with basophils through a calcium-fluxing receptor inducing antimicrobial, opsonizing, inflammatory, and immunostimulating factors (39). Equivalent cells only expressing IgD on the cell membrane (IgD^+^ B cells) have been reported in channel catfish (*Ictalurus punctatus*) blood (40) and rainbow trout (*Oncorhynchus mykiss*) gills and intestine (41, 42), and secreted IgD has been identified as well in many fish species including catfish, rainbow trout, Atlantic salmon (*Salmo salar*), Atlantic cod (*Gadus morhua*), and grass carp (*Ctenopharyngodon idella*) (40, 43). Interestingly, in rainbow trout, IgD secreted by these gut IgD^+^ B cells, has been shown to coat the intestinal microbiota (42) as described for teleost IgM and IgT (33), suggesting that secreted IgD may play an evolutionary conserved role in mucosal homeostasis (42), as previously suggested in mice (44).

Cellular immunity also plays a key role in mucosal protection against pathogens through the generation of memory T cell subsets localized in mucosal tissues (45). For instance, in mice, an intratracheal vaccination strategy that combined Toll-like receptor (TLR) agonists with antigen-carrying lipid nanocapsules generated long-lived memory T cells not only in lungs but also in the intestinal and vaginal mucosa (45). Similarly, the pulmonary administration of a plasmid DNA vaccine induced robust systemic CD8^+^ T-cell responses in lungs and draining lymph nodes of mice equivalent to those generated by intramuscular vaccination (46). Recently, it has been shown in mice that the protective effect exerted by the intranasal administration of a recombinant murine cytomegalovirus expressing an MHC I restricted epitope from influenza A virus H1N1 was critically mediated by antigen-specific CD8^+^ T cells (47). In fish, a high number of T cells have been reported to be present in mucosal surfaces such as intestine and gills in several fish species and the role of CD8^+^ T cells mediating cellular protection against intracellular bacteria or viruses has been established [reviewed by Nakanishi et al. (48)]. However, to our knowledge, information regarding how T cells locally respond to mucosal vaccination in fish is missing.

Taking all these specific features of the fish immune system into account, the characterization of how mucosal immune cells respond to antigens locally, and then deliver activation signals to systemic immune tissues has special relevance to find out which type of mucosally delivered vaccine formulations are capable of providing strong and long-lasting B and T cell memory responses in these species. Consequently, we have focused this work on reviewing the adaptive immune responses elicited in fish upon mucosal immunization through different routes of administration including oral and nasal vaccination, anal intubation, and immersion vaccination. We have also highlighted the gaps found to evaluate T and B cell responses for some specific vaccines and routes of administration. Specifically, we have considered those studies in which at least one of the following responses has been reported: antibody response in serum, transcriptomic effect in genes related to adaptive immune response (*e.g.*, IgM, IgT, IgD, MHC II, CD4, and CD8), as well as T cell-mediated immunity and B cell responses observed in mucosal and also in central tissues as thymus, peripheral blood, head kidney, and spleen. Finally, taking all this information into consideration, we discuss several strategies to improve the efficiency of mucosal vaccination in teleost fish.

## Local and Systemic Effects After Oral Vaccination

Several studies have been focused on evaluating the local and systemic effects of oral immunization against bacteria and viruses in fish since this route of administration requires less fish handling, induces minimum stress, reduces side effects, and also allows vaccination at any stage of the life cycle in fish (49). Oral immunization of antigens can be administered by a great variety of ways including non-encapsulated or encapsulated feed, gavage (considered as an experimental way of delivering vaccines) or through the use of bioencapsulation vectors such as planktonic *Crustacea* (*e.g.*, *Daphnia*), rotifers, and *Artemia*, all of them aimed at protecting the antigen from degradation until it reaches the most posterior segments of the digestive tract where immune induction has been shown to take place (21). These include the hindgut (50, 51) as well as the pyloric caeca, characterized in rainbow trout to be one of the most responsive gut segments to immunization (52).

### Antibacterial Vaccines

Some vaccines designed against bacterial pathogens have been shown to produce high levels of serum antibodies when administrated in the fish feed (53–57). For instance, the level of protective antibodies elicited by a live attenuated *Streptococcus agalactiae* vaccine in Nile tilapia (*Oreochromis niloticus*) was significantly higher compared to the control group, reaching their maximum levels at 14–21 days after the immunization (55). In the case of the red tilapia hybrid (*Oreochromis mossambicu*s x *O. niloticus*), fish vaccinated with formalin-killed *St. agalactiae* showed significantly higher IgM levels compared to controls, lasting up to 6 weeks after administration of the first booster dose (54). However, when a double booster regime was undertaken consistent higher levels of antibodies were reached until week 12, suggesting that this feed-based vaccination regime is a suitable strategy to obtain a long-lasting protection (54). Similarly, rainbow trout immunized with a non-microencapsulated live rifampicin-attenuated oral vaccine against *Flavobacterium psychrophilum* reached higher antibody titers than those obtained in fish vaccinated with the equivalent microencapsulated vaccine (53). Interestingly, a similar antibody response was observed between fish vaccinated orally and those intraperitoneally injected with the non-encapsulated vaccine (53). In the same way, oral vaccination of Atlantic salmon (*S. salar*) with live attenuated *Piscirickettsia salmonis* using a bioadhesive MicroMatrixTM cationic polysaccharide formulation produced systemic anti-*P. salmonis* IgM with a magnitude slightly higher than that of sera obtained from intraperitoneally vaccinated fish (57). In this study, specific antibodies were also detected in the intestinal mucosa of the oral vaccine-treated group at 350 degree-days post-vaccination (57).

In addition to these studies that show that some oral vaccination strategies are capable of producing systemic in addition to mucosal antibodies (57), there are very few data available on the characterization of B and T cell responses in central or mucosal tissues after oral immunization by means of transcriptional or functional analysis. In this sense, a study performed in gilthead sea bream (*Sparus aurata*) showed that the levels of specific IgM antibodies in the serum and IgT antibodies in skin mucus were significantly increased in orally vaccinated animals when challenged with *Photobacterium damselae* subsp. *piscicida* (37). In this case, challenged fish that had been orally vaccinated showed increased transcription of secreted IgT in intestine and head kidney, and much higher in spleen when compared to challenged unvaccinated fish (37). Regarding IgM, the transcription of membrane IgM was significantly upregulated in spleen, whereas the transcription of both membrane and secreted IgM forms were significantly downregulated in the intestine (37).

As an alternative to being a route for vaccination *per se*, oral vaccination has been proposed as a booster to acquire longer protection to intraperitoneally administered antigens. In this sense, the production of specific IgM antibodies was significantly increased when Atlantic salmon, rainbow trout and coho salmon (*Oncorhynchus kisutch*) were boosted orally after having been vaccinated with an injectable mono or polyvalent vaccine against salmonid rickettsial septicemia (SRS) (56). The oral boosters not only increased the magnitude of the response but also maintained the antibody response for longer, up to 2800–3200 degree-days (56).

### Antiviral Vaccines

The development of oral vaccines against viruses has been the aim of many studies in recent years (49, 50, 52, 58–63). Some of these studies have shown that oral antiviral vaccines can increase both systemic and mucosal immune responses to a previously administered intraperitoneal vaccine. Specifically, the administration of alginate-encapsulated antigens of infectious pancreatic necrosis virus (IPNV) in the feed after intraperitoneal vaccination in Atlantic salmon induced not only a significant higher antibody response following the first and the second booster, but also a significant higher CD4 and GATA3 expression in head kidney and spleen, known to be associated with T helper 2 responses that induce the secretion of Igs by B cells (61). Additionally, IgT transcription was also significantly increased in the intestine of fish orally immunized with the alginate-encapsulated vaccine (61). In some other cases, the antiviral vaccine was capable of inducing a strong immune response on its own. For example, in rainbow trout, detectable levels of anti-IPNV neutralizing antibodies were observed 90 days after administering along with the feed, for three consecutive days, 10 µg of an alginate-encapsulated DNA vaccine coding for the VP2 gene of IPNV (pcDNA-VP2) (59). Furthermore, IgM and IgT transcription was also significantly increased in response to this vaccine in kidney at day 30 post-vaccination (59). When this oral DNA vaccine was administered directly into the esophagus, neutralizing antibodies were also evident at 2, 3, 4, and 8 weeks post-vaccination (62). Additionally, IgM and IgT mRNA levels strongly increased in pyloric caeca, and to a lesser extent in head kidney (60). Accordingly, the pyloric caeca region was the area of the digestive tract in which a significant increase in the number of both IgM^+^ and IgT^+^ lymphocytes was more evident in response to this oral alginate-encapsulated IPNV DNA vaccine by means of immunohistochemistry analysis (52). Similarly, anti-IPNV antibodies were also detected in the sera of rainbow trout orally vaccinated with a DNA vaccine encapsulated in alginate microparticles as well as in chitosan/tripolyphosphate nanoparticles (58). In this case, the transcription levels of IgM, IgT, and CD4 (related to the presence of T helper cells) were dependent on the vaccine dose and were significantly upregulated in rainbow trout head kidney of all orally vaccinated fish groups compared to controls (58).

A significant increase in the production of antibodies against the infectious hematopoietic necrosis virus (IHNV) was detected in serum samples of rainbow trout vaccinated with an alginate microsphere encapsulated-DNA vaccine compared to sera from unvaccinated fish (50). Remarkably, this vaccine also induced the expression of several markers of the adaptive immune response, such as CD4, CD8, IgM, and IgT in the kidney and the spleen in a dose-dependent manner, although it should be noted that the vaccine doses that had to be used to achieve these responses were much higher than those used in the case of the oral alginate-encapsulated IPNV DNA vaccine (50). In 2017, a description of mucosal and systemic immune responses to IHNV induced by an orally delivered yeast expressing the G of IHNV on the cell surface was reported (63). In this work, the oral vaccine provoked a significant upregulation of CD8, IgM, and IgT mRNA levels in the hindgut, spleen, and head kidney from rainbow trout, with stronger levels reached in the intestine than in the other tissues (63).

The capacity of orally administered antiviral vaccines to induce antibodies has also been demonstrated in several species of carp (64–68). A single oral immunization using a formalin-inactivated crucian carp hematopoietic necrosis virus (CHNV) was not sufficient to induce measurable serum antibody responses in ginbuna crucian carp (*Carassius auratus langsdorfii*) (67). However, when fish were orally boosted 7 days after the initial oral immunization, significant antibody titers were reached between 30 and 60 days post-booster (67). Remarkably, orally immunized had higher antibody titers compared to non-immunized controls, and also significant cytotoxic peripheral blood lymphocytes (PBLs) against fibroblastic target cells fish and intraperitoneally infected when challenged with the live virus (67). In another study, VP6-specific serum antibodies were detected in grass carp (*Ctenopharyngodon idella*) during 2–8 weeks after vaccination with a recombinant baculovirus containing the *vp6* gene from grass carp reovirus (GCRV) (68). Similarly, when common carp (*Cyprinus carpio*) and koi (*C. carpio koi*) were orally immunized with a recombinant *Lactobacillus plantarum* expressing the G protein of spring viremia of carp virus (SVCV) combined with the ORF81 protein of koi herpesvirus (KHV), significant levels of specific IgM were detected against the two viruses (64). Following a different approach, when common carp (*C. carpio* var. Jian) larvae were fed with *Artemia* coated with recombinant *Saccharomyces cerevisiae* expressing the cyprinid herpesvirus-3 (CyHV-3) envelope antigen pORF65 high levels of specific anti-pORF65 antibodies were induced, in addition to higher levels of IgM and CD8α transcription in the spleen (66). Similarly, the oral gavage of *S. cerevisiae* expressing CyHV-3 ORF131 evoked the production of specific serum IgM as well as the increase of the IgT1 mRNA levels in the spleen and the head kidney (65). However, no differences were observed between carps immunized with vaccine and the control yeast, attributing the increase of the IgT expression to the mucosal modulator of the yeast rather than to the expressed protein in this microorganism (65).

## Local and Systemic Effects After Immersion Vaccination

There are two methods to immunize fish by immersion: i) dip vaccination in which fish are immersed in water containing a relatively high dose of vaccine for few seconds or minutes; and ii) bath vaccination in which fish are introduced in a more diluted vaccine solution for a longer period of time (69). Although the great advantage of immersion vaccination is that the antigen is taken up through the skin, the intestine and gills, very large amounts of antigen are required and usually the levels of protection reached are not high, possible due to the fact that only a limited amount of antigen reaches the immune effector sites in these mucosal tissues (70). Despite this, being a method suitable to vaccinate a large number of fish at the same time, it has been used to administer many commercial antibacterial vaccines for fish. Commercial immersion vaccines commonly used in aquaculture include vaccines such as that against pasteurellosis for sea bream or against vibriosis for sea bass (*Dicentrarchus labrax*) and turbot (*Scophthalmus maximus*).

### Antibacterial Vaccines

Most studies focused on establishing the local and systemic effects of immersion vaccines has been carried out with antibacterial vaccines, specifically against *Yersinia ruckeri*, *F. psychrophilum*, *Listonella* (formerly *Vibrio*) *anguillarum*, and *Edwardsiella tarda*. In most of these studies, except for those performed with *E. tarda*, the vaccines were administered by the dip methodology. Thus, dip vaccination of rainbow trout against enteric red mouth disease (ERM) using a commercial *Y. ruckeri* bacterin biotype 1 (AQUAVAC ERM vaccine, MSD Animal Health) (1:10 diluted-vaccine for 5 min) has shown to significantly increase *Y. ruckeri-*specific IgM antibody titers at 4, 8, and 26 weeks post-vaccination (71). Similarly, a booster vaccination using different dilutions (1:100 and 1:1,000) of the commercial vaccine AQUAVAC RELERA (MSD Animal Health) which combines *Y. ruckeri* biotypes 1 and 2, significantly increased antibody levels compared to fish vaccinated with only one dose (72). Interestingly, both commercial vaccines when administered by dip vaccination (1:10 diluted-vaccine for 30 s) increased the expression of adaptive immune genes in the later phase of infection, and also the occurrence of CD8^+^ and IgM^+^ cells in the spleen and head kidney, thus correlating the cellular changes with protection of vaccinated fish (73).

Significant levels of *F. psychrophilum*-specific antibodies were also reported in dip vaccinated rainbow trout (1:10 diluted-vaccine for 3 min) in several studies (70, 74). In addition, the administration of a polyvalent whole cell vaccine containing formalin-inactivated *F. psychrophilum* (1:10 diluted-vaccine for 30 s) elicited a low, although significant, increment of serum IgT at 6 weeks post-vaccination in rainbow trout (75). In this study, the number of IgT^+^ cells detected in spleen and head kidney of immersion vaccinated fish was also significantly higher in vaccinated fish. Intriguingly, the expression of CD8α in the head-kidney at day 2 and the levels of CD4-1 and CD8α transcription in the spleen at day 7 were significantly downregulated in vaccinated fish, suggesting the mobilization of both helper and cytotoxic T cells from these organs. However, IgT and IgM transcription levels were significantly upregulated in the hindgut at day 2 (75).

Dip vaccination of sea bass with a whole killed *L. anguillarum* serotype O1 and O2 bivalent commercial vaccine (MICROViB, Microtek International) also produced an increase of specific IgM antibodies (76). In zebrafish (*Danio rerio*) dip vaccinated for 8 min with attenuated *L. anguillarum*, the specific antibody levels did not rise significantly following vaccination (77). A recent study analyzed the response of Atlantic cod (*G. morhua*) to a dip vaccination against *L. anguillarum* (1:10 diluted-vaccine for 30 s), revealing changes in the levels of transcription of genes related to adaptive immunity processes as established by means of a transcriptome analysis in head kidney (78). Thus, genes related to T cell activity (CD8, TRGC1, TNFSF11, EOMES, PERF1, UNC13D), and B cell activity (IGLC2) were positively differentially expressed in vaccinated fish (78). Specifically, TRGC1 is a T cell receptor gamma and TNFSF11 is involved in the regulation of T cell-dependent immune responses. On the other hand, the increase in EOMES, PERF1, and UNC13D levels observed in vaccinated fish suggest the specific activation of cytotoxic T cells that preferentially express these genes (78).

Regarding the immune response elicited by vaccines administered by bath, the immunization of Japanese flounder (*Paralichthys olivaceus*) with a formalin-inactivated form of *E. tarda* produced a significantly higher upregulation of MHC II, TCRα, CD4-1, and IgT transcription in the skin and gills than that observed in spleen and kidney (79). On the contrary, the expression of IgM was much higher in lymphoid organs than in mucosal tissues (79).

### Antiviral Vaccines

The efficacy of antiviral vaccines administered by bath to induce B and T cell responses has been also demonstrated in fish (80–82). For instance, a significant specific antibody response was detected in carp 28 and 36 days after bath vaccination for 30 min with a KHV vaccine which consisted in inactivated *Escherichia coli* expressing glycoprotein-25 (80). In another study, carbon nanotubes were tested as vaccine carriers to enhance the efficacy of an immersion vaccine (81, 82). Specifically, functionalized single-walled carbon nanotubes (SWCNTs) used as carriers of a DNA vaccine encoding a matrix protein of SVCV and expressing a green fluorescent protein (pEGFP-M) produced a significant increase of antibody levels and an upregulation of IgM, IgZ1, CD8β, CD4, MHC I, and MHC II transcription levels in carp immunized by bath twice for 10 h after a 3 day interval (81), suggesting the activation of both cytotoxic and helper T lymphocytes upon immunization. Similarly, bath vaccination of Mandarin fish (*Siniperca chuatsi*) for 10 h with a subunit vaccine system encoding the major capsid protein (MCP) of the infectious spleen and kidney necrosis virus (ISKNV) based on SWCNTs produced higher levels of antibody as well as higher IgM mRNA levels than those obtained in groups vaccinated with the naked vaccine (82).

## Local and Systemic Effects After Nasal Vaccination

After the identification of the nasal-associated lymphoid tissue (NALT), some studies have emphasized the importance of nasal immunity to both infections and intranasal immunizations (83). In this respect, the efficacy of nasal vaccines has been uniquely demonstrated in rainbow trout (25, 84, 85), and as a consequence, only a few studies have identified the mucosal and systemic mechanisms elicited by vaccines administered through this route (23, 25).

### Antibacterial Vaccines

There is only one study focused on how fish respond locally and systemically to an antibacterial vaccine administered through the nasal cavity (23). In this study, an inactivated vaccine against *Y. ruckeri* administered either nasally or intraperitoneally in rainbow trout produced an increase of the nasal IgM repertoire diversity, indicating that both local and systemic antigen exposures are capable of inducing B cell responses in the NALT (23). In the case of splenic B cells, nasal vaccination decreased both IgM and IgT clonotype diversity. On the other hand, specific serum IgM and IgT titers were significantly risen after intraperitoneal vaccination but not after nasal immunization (23).

### Antiviral Vaccines

Concerning antiviral vaccines, the administration of a live attenuated IHNV vaccine into the olfactory cup, was reported to elicit an antiviral adaptive immune response in the head kidney and the olfactory organ similar to that triggered by IHNV intramuscular vaccination (25). Moreover, the expression of IgM mRNA levels were significantly higher in the olfactory tissue at day 14 post-immunization in comparison to day 4, indicating the capacity of this vaccine to induce an adaptive immune response (25).

## Local and Systemic Effects After Anal Intubation

Some studies have explored the effects of anal immunization. Although this immunization strategy offers many disadvantages from a practical point of view, it can be considered as a way to investigate how the intestinal mucosa directly reacts to antigens without having to protect the antigen from degradation in the upper digestive segments (86, 87). In 2000, a study addressed how rainbow trout responded to fluorescein isothiocyanate (FITC) conjugated to keyhole limpet hemocyanin (KLH) when was administered intraperitoneally or anally (88). The results showed that fish injected with FITC-KLH developed higher antibody level of mucosal and serum anti-FITC antibodies when compared to fish immunized anally (88). Recently, the anal route has been used to study specific B cell responses to two model antigens, TNP-KLH (2,4,6-trinitrophenyl KLH), a TD antigen and TNP-LPS, a TI antigen (87). This study demonstrated that, in the absence of additional adjuvants, rainbow trout preferentially responded to anally administered TNP-LPS, while the response to TNP-KLH was much weaker. Thus, TNP-LPS elicited TNP-specific serum IgM, and a significant increase in the number of total and TNP-specific IgM-secreting cells in both spleen and kidney, with the kidney being the site where most of these cells were found at later time points. In the spleen, a proliferative response of both IgM^+^ B and T cells was also clearly visible at early time points, while weaker proliferative responses were observed in the kidney. At transcriptional level, a significant decrease in FOXP3A, FOXP3B, CD4, and CD8 mRNA levels was detected in the intestine of TNP-LPS-immunized fish, which suggested a local downregulation of different T cell subsets (87).

### Antibacterial Vaccines

Few data is available concerning the effect of antibacterial vaccines administered anally. A *L. anguillarum* inactivated vaccine as well as the extracellular products obtained when producing the vaccine were shown to increase antibody titers in carp 21 days after anal intubation (89).

### Antiviral Vaccines

A formalin-inactivated CHNV administered by anal intubation produced a significant upregulation of TCRß mRNA levels in the kidney and the intestine of ginbuna crucian carp when compared to control unvaccinated fish (90). This result led the authors to hypothesize that this vaccine was effective at triggering systemic and local T cell responses. Additionally, an increase of the percentage of proliferating CD8^+^ cells was detected in the posterior portion of the hindgut, pointing to this part of the intestine as an important site for generating virus-specific CD8^+^ cytotoxic T cells upon anal vaccination (90).

## Comparison of The Local and Systemic Effects After Immunization by Several Routes

In some studies different routes of mucosal administration have been compared in order to identify which delivery method reached the higher adaptive immune responses at both local and systemic levels.

### Antibacterial Vaccines

In 1986, a study performed in carp showed that a formalin-killed *L. anguillarum* vaccine administered by anal immunization increased antigen-specific antibodies in serum, and after anal boosting, similar levels of serum antibodies were reached compared with two consecutive intramuscular injections (91). An enhancement of antigen-specific Ig titers in skin mucus was also detected after anal immunization (91). However, no significant antibody titers were elicited in serum after oral immunization, not even when bacteria were administered daily with the food (91). In rainbow trout, protection against *L. anguillarum* correlated positively with serum antibody levels after, but not prior to, boosting by oral, immersion or injection with a formalin-killed vaccine (92). In the case of African catfish (*Clarias gariepinus*), the analysis of serum samples after vaccination with a formalin-inactivated *L. anguillarum* O2 bacterin by four delivery routes showed that fish vaccinated by intraperitoneal injection obtained the highest antibody levels, followed by fish immunized by anal intubation, oral administration and immersion (93). However, the levels of antibodies reached in skin mucus were higher upon anal intubation, followed by immersion, oral intubation, and intraperitoneal injection (93), demonstrating that mucosal vaccines have a superior capacity to induce mucosal responses. In the case of barramundi (*Lates calcarifer*), fish injected intraperitoneally with an experimental *Vibrio harveyi* inactivated vaccine displayed significantly higher serum antibody activity than immersed and intubated fish (94). In eel (*Anguilla anguilla*), the bivalent formalin and heat-inactivated vaccine against two eel-pathogenic serovars of *Vibrio vulnificus* induced the production of significant antibody levels in plasma as well as in the skin and gut mucus despite the *via* of administration (oral, anal, immersion, or intraperitoneal vaccination) (95). Interestingly, in all cases, the production of IgM was faster in mucus while antibody titers were higher and lasted longer in plasma (95). In rainbow trout, no significant increase of IgM titers in serum, gill or skin mucus was observed 28 days after immunization of fish with live attenuated *F. psychrophilum* by anal intubation or immersion, unlike fish vaccinated intraperitoneally (96). However, the levels of secretory IgD and IgT expression were significantly upregulated in gills and intestine of fish immunized by the immersion and anal intubation route, respectively (96). In another study, the oral or anal administration of rainbow trout with a *Y. ruckeri* O1 bacterin induced protection against ERM, without affecting the levels of *Y. ruckeri* specific antibodies in plasma. The authors attributed this protection to an immune response mounted locally in the intestine (97). On the contrary, significantly higher antibody levels in serum were detected 45 days post-immunization in mrigal (*Cirrhinus mrigala*) anally or orally vaccinated against *E. tarda* in comparison to those detected in control fish or in fish vaccinated by immersion and intraperitoneal injection (98). In skin and gill mucus, significantly higher antibodies levels were obtained in the oral and immersion groups (98). In gut mucus, significantly higher values of antibodies were detected in the immersion group compared to the rest of the treatments (98). However, oral and immersion immunization routes offered better protection of mrigal compared to other antigen delivery routes (98).

### Antiviral Vaccines

No significant differences were observed in serum antibody levels in rainbow trout intraperitoneally, anally, and orally immunized with viral hemorrhagic septicemia virus (VHSV) (99). The vaccine consisted in lyophilized virus surrounded by polyethylene glycol (PEG) and then extruded under low temperature (99). In olive flounder fingerlings, specific anti-VHSV antibody titers were significantly enhanced in serum, and also in the skin and intestinal mucus following vaccination with a poly lactic-co-glycolic acid (PLGA) or chitosan encapsulated formalin-inactivated VHSV vaccine administered together with the feed or by immersion (100, 101). Additionally, a significant upregulation of IgM, IgT, pIgR, MHC I, MHC II transcripts was detected in the kidney, skin, and intestine of vaccinated fish (100, 101).

Bath and oral immunizations of grouper (*Epinephelus coioides*) larvae with a binary ethylenimine (BEI)-inactivated nervous necrosis virus (NNV) vaccine induced the transcription of IgM, IgT, MHC I, MHC II, and CD8 not only in viscera but also in mucosal tissues at specific time points (102), suggesting the involvement of both local and systemic B and T cells.

## Strategies for Improving The Efficiency of Mucosal Vaccination in Teleost Fish

There are many mucosally administered vaccines that are only capable of triggering local immune responses and have been shown to be incapable of providing fish with a sufficient level of protection upon pathogen encounter [reviewed in (21)]. Thus, although in many of the studies mentioned through this work the levels of protection conferred by the vaccines have not been studied, it seems clear that those mucosal vaccines that are capable of eliciting strong systemic B and T cell responses are better suited to confer protection, and these are the ones we have focused on in this paper. However, it should be noted, that most of these studies in which adaptive immune responses to vaccines were undertaken, antibody production was measured and very few of them analyzed T cell responses, despite their relevance, especially in the case of antiviral vaccines. Additionally, all determinations of specific antibody titers focused on the identification of IgM specific antibodies, as no studies to date have reported the presence of antigen-specific IgT or IgD antibodies in fish. Therefore, the development of additional immunological tools in different fish species that would allow us to have a wider view of how B and T cell responses are activated in response to vaccine antigens seems essential to establish immunological correlates of protection. Only through a complete understanding of how antibodies and cellular responses correlate to protection, we will be able to rationally design effective mucosal vaccines in the future.

To increase the efficacy of non-optimal mucosal vaccine formulations, two aspects have to be taken into account. The formulation of the antigen has to be optimized to allow it to reach mucosal effector sites in an unaltered way in sufficient amount. For oral vaccines, this implies reaching the more distal gut segments such as hindgut (50) or pyloric caeca (52). However, knowledge on how the antigen is taken up at these mucosal sites and presented to cells of the adaptive system is still scarce. These studies are essential to understand to which cell types the antigen should be directed. For oral vaccines, encapsulation has been the most commonly used method to protect antigens from degradation and increase their uptake in the gut after oral immunization, whereas a fewer number of studies have used alternative strategies such as antigen expression in live feed (58). The other aspect that should be taken into account to increase the efficacy of mucosal vaccines, is the fact that these vaccines should circumvent mucosal tolerance, a mechanism through which the immune response in these mucosal surfaces is tightly regulated to avoid a continuous response to innocuous antigens. To this aim, inclusion of adjuvants may help to increase the immunogenicity of these antigens and bypass mucosal tolerance. However, this implies a greater knowledge of these regulatory systems as well as a search for adjuvants suited for mucosal deliver in fish. For instance, in mammals, the most commonly used mucosal adjuvants are toxin-based adjuvants (*e.g*., enterotoxigenic *E. coli* heat-labile toxin and cholera toxin), immunostimulatory adjuvants (*e.g.*, monophosphoryl lipid, CpG, and QS21, a substance extracted from the bark of *Quillaja saponaria*), particulate adjuvants (*e.g*., emulsions and highly immunogenic immune stimulating complex) (103), or microbiota-derived components such as flagellin (104). Very few information is available regarding the adjuvant potential of these molecules in fish. The adjuvant potential of the *E. coli* LT (R192G/L211A) toxoid (dmLT) was first tested in carp in combination with an oral DNA vaccine against SVCV showing no efficacy (105). However, taking into account that a specific adjuvant may not be suitable for one antigen, but could be effective in combination with one of a different nature, Martín-Martín et al. (106) established a simple protocol designed to initially evaluate the effects of potential oral adjuvants for two different pathogens. The results pointed to dmLT as an preferential adjuvant for oral antibacterial vaccines in rainbow trout, since the combination of the toxoid with VHSV only produced minor transcriptional effects, while the effects were much more pronounced when combined with *Aeromonas salmonicida* (106). The adjuvant effect of *Q. saponaria* saponin (QSS) has been also tested in fish, particularly on the protection of turbot fry against *L. anguillarum* (107). The results showed that turbot immersed in seawater containing QSS for 10 min and then vaccinated in a formalin-inactivated *L. anguillarum* MN suspension for 30 min produced a significant expression of IgM mRNA in skin, spleen, and kidney at 14 days post-immunization in comparison to fish vaccinated without QSS by immersion or intraperitoneal injection (107). Moreover, a higher serum antibody titer was also demonstrated after 14 days in the vaccinated fish although at levels lower than those reached in the intraperitoneally immunized fish (107). Another strategy which has been proposed for aquacultured fish is the use of proteins derived from the host rather than foreign antigens as adjuvants (108). This is the case of sea bass fed with a commercial vaccine against *L. anguillarum* and *Vibrio ordalii* (AQUAVAC Vibrio Oral, ISPAH) adjuvanted with a fish-self recombinant cytokine, such as the tumor necrosis factor α (rTNFα). The supplementation of this vaccine with rTNFα significantly enhanced disease resistance against vibriosis, enhancing the infiltration of T cells into the gut epithelium, upregulating the expression of the IgT gene in the hindgut but not raising specific IgM titers in serum (108).

Regarding microbiota-derived components, the immune-stimulating effects of β-glucans on the fish immune system when administered on their own have become evident using different administration routes (109). Recently, immersion vaccination of gilbel carp (*Carassius auratus gibelio*) with β-glucans and β-propiolactone-inactivated cyprinid herpesvirus 2 protected against a viral challenge more efficiently than fish vaccinated in the absence of β-glucans (110). In this work, the levels of transcription of IL-2 and IFN-γ2 in head kidney and spleen as well as of IgM and IgZ in the spleen were higher when β-glucans were included (110). In rainbow trout, a recombinant flagellin from the salmonid pathogen *Y. ruckeri* induced a strong antibody response when intraperitoneally injected (111). Thus, it would be interesting to study how fish B and T cells respond to bacterial flagellin when administered as a mucosal adjuvant and whether this protein is able to promote adaptive immune responses in fish as previously observed in mice (104).

It is worthwhile to mention that, in the last years, not only microbiota-derived components but also live microorganisms, designated as immunobiotics, have been shown to modulate the mucosal immune system. Therefore, immunobiotics have been considered as a potential alternative to enhance the response to vaccination (112, 113). For example, the nasal administration of *Lactobacillus rhamnosus* CRL1505 as an adjuvant improved the humoral and cellular adaptive immune responses induced by influenza virus infection or vaccination in mice (112). Regarding this strategy, few data have been reported in fish. Yeast has been proposed as vehicle for oral antigen delivery due to its immunomodulatory properties and potential to boost local immune responses, acting as an adjuvant (49). Thus, in rainbow trout, the oral administration of *Pichia pastoris* yeast expressing a green fluorescence protein (GFP) was shown to exert a rapid local innate immune response in the intestine increasing IgT transcription in the midgut at 24 h post-treatment, and a subsequent systemic response, increasing the transcription of IgM and IgT in the spleen after 7 days (49). In this scenario, it is plausible that the use of immunobiotics can be explored in the future as adjuvants for novel fish mucosal vaccine formulations.

## Concluding Remarks

Taking into account that pathogens enter the organisms through mucosal surfaces where they initiate their replication and having established that mucosal vaccines have a superior ability to trigger mucosal immunity than systemic vaccines, mucosal vaccines seem as a great alternative to vaccines administered through injection. However, it does seem that these vaccines must be capable of eliciting robust systemic immune effects in order to provide long-lasting memory B and T cell responses. Although in fish, mucosal vaccines also provide numerous advantages from a practical point of view, almost no oral vaccines and very few immersion vaccines are available in the market for aquacultured species, many of them intended as boosters and not as vaccines on their own. The efficacy of these vaccines depends not only on the immunogenicity of the antigen itself (that may be increased by the addition of suited adjuvants) but also on the availability of this antigen for mucosal immune cells. In this sense, in mammals and also in fish, recent strategies to develop effective mucosal vaccines have been aimed at directing the antigens to specific antigen sampling cells within the mucosa (10, 114, 115). However, these strategies require a much better knowledge of how immune responses are organized in mucosal surfaces in fish, as well as a full phenotypical and functional characterization of the cells implicated in this response. Thus, the design of efficient mucosal vaccines for fish still requires investigation of many aspects. In parallel, it is also essential to develop new immunological tools that can help us evaluate the response to these vaccine formulations and establish correlates of protection. Despite all these gaps, it seems obvious that those mucosal vaccines capable of inducing not only local, but also systemic B and T cell responses are better placed to confer high protection levels. Thus, in this review we have highlighted this information, as it could be used as a starting point to continue the optimization towards economically profitable mucosal vaccines.

## Author Contributions

EM-A collected all the information included in this review and wrote the manuscript with help and contributions from PD-R and CT. All authors contributed to the article and approved the submitted version.

## Funding

This work was supported by the European Research Council (ERC Consolidator Grant 2016 725061 TEMUBLYM), by the Spanish Ministry of Science, Innovation, and Universities (project AGL2017-85494-C2-1-R) and by the *Comunidad de Madrid* (grant 2016-T1/BIO-1672). EM-A was recipient of a Juan de la Cierva-Incorporación Postdoctoral Contract (IJC2018-035843-I) funded by the Spanish Ministry of Science, Innovation and Universities.

## Conflict of Interest

The authors declare that the research was conducted in the absence of any commercial or financial relationships that could be construed as a potential conflict of interest.
